# Associations between food-specific IgG and health outcomes in an asymptomatic physical examination cohort

**DOI:** 10.1186/s12986-022-00657-5

**Published:** 2022-03-19

**Authors:** Mingxia Wu, Xiaofang Wang, Li Sun, Zongtao Chen

**Affiliations:** Health Management Center, First Affiliated Hospital of Army Medical University (Third Military Medical University), Chongqing, 400038 China

**Keywords:** Food-specific IgG, Physical examination cohort, Health outcomes, Body weight, Triglycerides, Fasting blood glucose

## Abstract

**Background:**

Although the association of food-specific IgG with the development and progression of specific diseases was shown by many studies, it is also present in the population without clinical symptoms. However, the association between food-specific IgG and physical examination outcomes in healthy people has not been studied yet.

**Methods:**

An asymptomatic physical examination cohort (APEC) was selected according to the inclusion and exclusion criteria, the physical examination data were compared between IgG positive and IgG negative groups, and their odds ratios (ORs) and 95% confidence intervals (CIs) were calculated using multivariable logistic regression.

**Results:**

The data of 28,292 subjects were included in the analysis. The overall IgG positive rate was up to 52.30%, mostly with mild to moderate IgG positivity. The multivariable Logistic regression showed the prevalence of hypertriglyceridemia, abnormal fasting blood glucose and overweight was lower in the IgG (+) positive group (OR 0.87, 95% CI 0.83–0.92; OR 0.93, 95% CI 0.87–0.99; OR 0.92, 95% CI 0.87–0.96) but there was a higher prevalence of thyroid disease (OR 1.09, 95% CI 1.04–1.15).

**Conclusion:**

Food-specific IgG positivity was widespread in the APEC and was associated with lower prevalence of hypertriglyceridemia, abnormal fasting blood glucose and overweight. The underlying physiological mechanism merits further study.

## Introduction

At present, the effects of food-specific IgG on human health remain controversial. Numerous studies have suggested that food-specific IgG is involved in the development and progression of specific diseases, such as inflammatory bowel disease [1], irritable bowel syndrome [2], migraine [3, 4] and mental disease [5–7]. The symptoms of these disease can be relieved by food-specific IgG-based diet recommendations [8–13]. IgG antibody can form an immune complex with allergens in foods and thus induce mild inflammatory reactions in the body [14], which are manifested as various systemic symptoms and diseases. According to other studies, however, food-specific IgG actually provides natural protection against food allergy [15, 16]. The most direct evidence comes from an oral immunotherapy (OIT) study showing that OIT for peanuts can induce a remarkable increase in the plasma levels of food-specific IgG [16–18], which indicates the involvement of IgG in mitigating the symptoms and inducing food tolerance of allergic patients.

Previous clinical studies on food-specific IgG typically focused on patients with a specific disease. Studies have found a high prevalence of serum IgG antibodies against specific food allergens in patients with IBD. Accordingly, IgG antibodies may potentially indicate disease status and be utilized to guide dietary recommendations for patients [19–21]. Additionally, migraine patients with positive food specific IgG antibodies had worse migraine, anxiety, and gastrointestinal symptoms [22]. Moreover, significantly higher serum food antigen-specific IgG positivity rates were found in patients with depressive disorder [23]. Food-specific IgG is also present in healthy subject [5], but this important has not received much attention. If food-specific IgG is associated with the development and progression of diseases, it is also important to analyze if it has correlates in the physical examination results of healthy subjects, which we investigated in this study.

## Methodology

### Study design and participants

The study data was based on a healthy APEC who firstly received the test of 14 food-specific IgGs at the Health Management Department of the First Affiliated Hospital of Army Medical University during 2010–2020 and met the following criteria: age ≥ 18 years, no previous food intolerance test, no food intake restriction, and no food allergy symptoms or resultant medical care requirement. Datasets without such information as age, sex, height and body weight were excluded. Other physical examination data were also collected, including blood pressure, waist circumference, hematological examination indicators (e.g., total cholesterol (TC), triglycerides (TG), low density lipoprotein cholesterol (LDL-C), high-density lipoprotein cholesterol (HDL-C), fasting blood glucose (FBG), uric acid (UA), plasma-albumin (ALB), liver function, hemoglobin and other examination items (e.g., thyroid ultrasonography, pulmonary radiography, abdominal ultrasonography, bone densitometry, gastroscopy, enteroscopy, and prostatic ultrasonography for men, as well as breast ultrasonography, uterus and appendage ultrasonography, as well as cervical cancer screening for women).

**Fig. 1:**
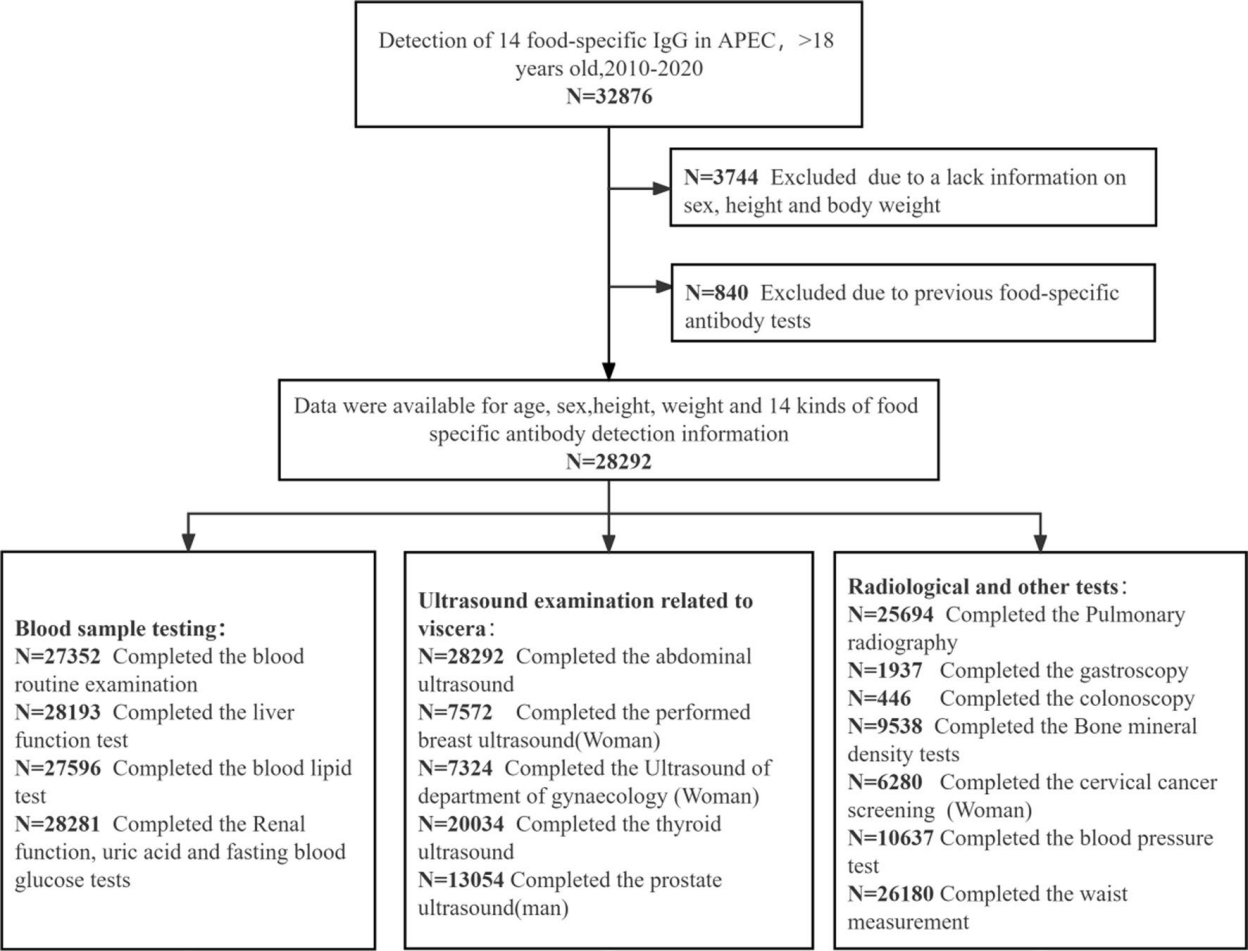
Flow chart of study population. (APEC, asymptomatic physical examination cohort; FBG, fasting blood-glucose. BP, blood pressure.)

After screening, a total of 28,292 physical examination subjects were included in the statistical analysis (32,876 subjects were preliminarily screened, 3744 of whom were excluded due to a lack information on sex, height and body weight, 840 were excluded due to previous food-specific antibody tests), and the various examinations they completed are shown in Fig. 1. The subjects were divided into two groups according to age (< 40 and ≥ 40 years). In these age groups, the probands were divided into IgG positive and IgG negative groups, and the above examination and test results were statistically analyzed (Table 1). To further investigate the relationship of food-specific IgG with the statistically different health outcomes, we established a model based on the available data. Specifically, we combined the influential factors of health outcomes reported previously, included factors such as age, sex, food-specific IgG, body weight, blood pressure, blood lipids, blood glucose, uric acid and fatty liver, and then calculated their OR and 95%CI using multivariable logistic regression. This study was approved by the Ethics Committee of the First Affiliated Hospital of Army Medical University (Approval No. KY2021046), and as all data were retrospectively analyzed in an anonymous form, the written informed consent from subjects was waived.

**Table 1:** Analysis of physical examination data among different subgroups

	< 40 year-old group				≥ 40 year-old group			
	IgG negative	IgG positive	t/x^2^	*P*	IgG negative	IgG positive	t/x^2^	*P*
*Gender*								
Woman (n/%)	1064/30.18	2055/42.50	132.47	< 0.001	3106/31.97	4442/43.47	280.73	< 0.001
Man (n/%)	2462/69.82	2780/57.50			6610/68.03	5773/56.53		
*Distribution of BMI*								
< 18.5 kg/m^2^ (n/%)	178/5.05	348/7.20	73.31	< 0.001	106/1.1	150/1.47	25.64	< 0.001
18.5–23.9 kg/m^2^ (n/%)	1596/45.28	2525/52.22			3632/37.37	4115/40.28		
≥ 24.0 kg/m^2^ (n/%)	1752/49.67	1962/40.58			5978/61.53	5950/58.25		
Central obesity (n/%)	973/29.93	1069/23.65	38.55	< 0.001	3611/40.34	3591/38.06	9.72	0.01
Dysarteriotony	181/12.03	163/9.54	5.22	0.02	1061/25.89	822/24.71	1.34	0.25
SBP (mmHg)	117.85 ± 15.52	115.88 ± 14.51	3.72	< 0.001	124.73 ± 18.64	124.63 ± 18.47	0.24	0.81
DBP (mmHg)	75.04 ± 11.86	72.42 ± 10.94	6.51	< 0.001	79.02 ± 12.94	78.28 ± 12.59	2.48	0.01
Dyslipidemia (n/%)	1751/49.66	2150/44.47	22.09	< 0.001	5769/59.37	5624/55.06	37.95	< 0.001
Hypertriglyceridemia (n/%)	1376/39.02	1390/28.75	97.25	< 0.001	4354/44.81	3882/38.01	95.24	< 0.001
Hypercholesteremia (n/%)	631/17.90	731/15.12	11.53	< 0.001	2785/28.66	2723/26.66	10.03	0.01
High LDL-C (n/%)	511/15.21	677/15.23	0.001	0.98	2391/24.78	2518/24.83	0.01	0.94
Low HDL-C (n/%)	181/5.39	175/3.94	9.23	0.01	443/4.59	412/4.06	3.34	0.07
*Distribution of FBG*								
< 6.1 mmol/L (n/%)	3241/91.92	4597/95.08	69.31	< 0.001	7902/81.33	8557/83.77	29.34	< 0.001
6.1–6.9 mmol/L (n/%)	181/5.13	158/3.27			1002/10.31	945/9.25		
≥ 7.0 mmol/L (n/%)	104/2.95	80/1.65			812/8.36	713/6.98		
Uric acid (umol/L)	364.05 ± 97.71	350.92 ± 98.28	6.05	< 0.001	353.57 ± 93.16	339.95 ± 93.75	10.28	< 0.001
Abnormal liver function (n/%)	1345/38.24	1374/28.58	86.27	< 0.001	3554/36.73	3151/30.92	74.86	< 0.001
Albumin (g/L)	46.98 ± 2.52	46.91 ± 2.55	1.39	0.17	45.52 ± 2.71	45.47 ± 2.73	1.34	0.18
Hemoglobin (g/L)	148.88 ± 15.61	145.02 ± 16.06	10.84	< 0.001	146.12 ± 15.55	142.98 ± 15.90	13.86	< 0.001
Abnormal renal function (n/%)	46/1.30	55/1.14	0.48	0.49	226/2.33	209/2.05	1.83	0.18
Fatty liver disease (n/%)	1114/31.59	1178/24.36	53.56	< 0.001	3826/39.38	3526/34.52	50.53	< 0.001
Gallbladder polyp (n/%)	274/7.77	328/6.78	2.97	0.09	928/9.55	901/8.82	3.19	0.07
Gallstone (n/%)	75/2.13	114/2.36	0.49	0.48	396/4.08	432/4.23	0.29	0.59
Renal calculus (n/%)	126/3.57	143/2.96	2.48	0.12	439/4.52	427/4.18	1.37	0.24
Abnormalbone mineral density (n/%)	373/69.63	373/64.42	3.42	0.06	3202/76.49	3209/75.81	0.54	0.46
Osteopenia (n/%)	297/55.0	285/49.22	3.74	0.05	1990/47.54	2016/47.63	0.01	0.94
Osteoporosis (n/%)	79/14.63	88/15.20	0.07	0.79	1212/28.95	1193/28.18	0.61	0.43
Thyroid nodule (n/%)	639/25.0	923/29.03	11.59	0.01	2849/39.67	3092/43.45	20.93	< 0.001
Lung lesions (n/%)	85/2.78	95/2.40	0.99	0.32	561/6.15	570/5.97	0.28	0.60
Gastric lesions (n/%)	60/28.04	69/27.49	0.02	0.90	296/38.99	268/37.69	0.27	0.61
Intestinal lesions (n/%)	8/18.61	7/15.91	0.11	0.74	52/27.08	42/25.15	0.17	0.68
Abnormal cervical liquid-based cytological test (n/%)	2/0.32	16/1.40	4.77	0.06	14/0.76	19/0.71	0.04	0.85
Breast lesions (n/%)	549/65.75	951/64.52	0.35	0.55	1323/60.99	1931/62.43	1.11	0.29
Abnormal gynecological ultrasonography (n/%)	273/36.45	491/38.69	1.01	0.32	1190/55.63	1809/57.72	2.26	0.13
Prostatic lesion (n/%)	76/6.11	59/5.01	1.40	0.24	1017/17.93	823/16.58	3.39	0.07

BMI, body mass index; SBP, Systolic blood pressure; DBP, Diastolic blood pressure; LDL-C, low density lipoprotein cholesterol; HDL-C, high density lipoprotein cholesterol; FBG, fasting blood-glucose

### Definition of positivity

In the present study, the food-specific IgG test was performed by detecting the serum IgG levels using an enzyme-linked immunosorbent assay (ELISA) and 14 foods most frequently eaten by the Chinese population (e.g., pork, rice, chicken, shrimp, corn, milk, soybean, wheat, mushrooms, beef, cod, crab, eggs and tomato). Four groups were divided according to IgG titers: negative (−, < 50 U/ml), mildly positive (+, 50–100 U/ml), moderately positive (++, 100–200 U/ml) and severely positive (+++, > 200 U/ml). Three groups were divided according to the number of IgG positive foods, negative (−), single food specific IgG positive (SIPO) and multiple foods specific IgG positive (MUPO).

Based on body mass index (BMI) (BMI = body weight/height^2^ (kg/m^2^)), the probands were classified into three types: underweight (BMI < 18.5 kg/m^2^), normal (18.5 kg/m^2^ ≤ BMI < 24.0 kg/m^2^) and overweight (BMI ≥ 24.0 kg/m^2^). The relevant definitions were as follows: adult central obesity, waist circumference ≥ 90 cm (for men) and ≥ 85 cm (for women); abnormal blood pressure, systolic blood pressure (SBP) ≥ 140 mmHg or diastolic blood pressure (DBP) ≥ 90 mmHg; abnormal blood lipids, any of TG > 1.73 mmol/L, TC > 5.7 mmol/L, LDL-C > 3.1 mmol/L and HDL-C < 0.9 mmol/L; abnormal FBG, FBG ≥ 6.1 mmol/L; hyperuricemia, UA > 428 µmol/L; abnormal liver function, alanine aminotransferase or aspartate aminotransferase > 42 IU/L; decreased ALB: ALB < 38 g/L; anemia: hemoglobin (HB) < 120 g/L (for men) and < 110 g/L (for women); abnormal renal function, blood creatinine (Cr) > 104 µmol/L; abnormal bone mineral density, including osteopenia (− 2.5 < T < − 1.0) and osteoporosis (≤ − 2.5); lung lesions, including pulmonary nodules, emphysema, lung cysts, lung cancer; gastrointestinal lesions, including gastrointestinal inflammation, polyps, ulcers, and cancer; breast lesions, including breast nodules, breast cysts, mammary duct lesions, and breast cancer; abnormal gynecological ultrasonography, including uterine and ovarian nodular lesions or cancer; abnormal cervical liquid-based cytological test, including low- and high-grade squamous intraepithelial lesions, and cervical cancer.

### Statistical analysis

The quantitative data of normal distribution were presented as means ± standard deviation (x ± S) and analyzed for pair-wise and multiple comparisons using independent samples *t*-test and analysis of variance (ANOVA), respectively. Data with a skewed distribution were expressed as medians and analyzed using the Kruskal–Wallis H test. Qualitative data were analyzed using the χ^2^ test. The OR and 95% CI were calculated using multivariable logistic regression to determine the relationship between food-specific IgG and different health outcomes. All statistical analyses in this study were conducted using SPSS software (v. 23.0; IBM Corp., USA). A *P*-value < 0.05 was considered to indicate statistical significance.

## Results

### Distribution profile of food-specific IgG in the APEC

**Fig. 2:**
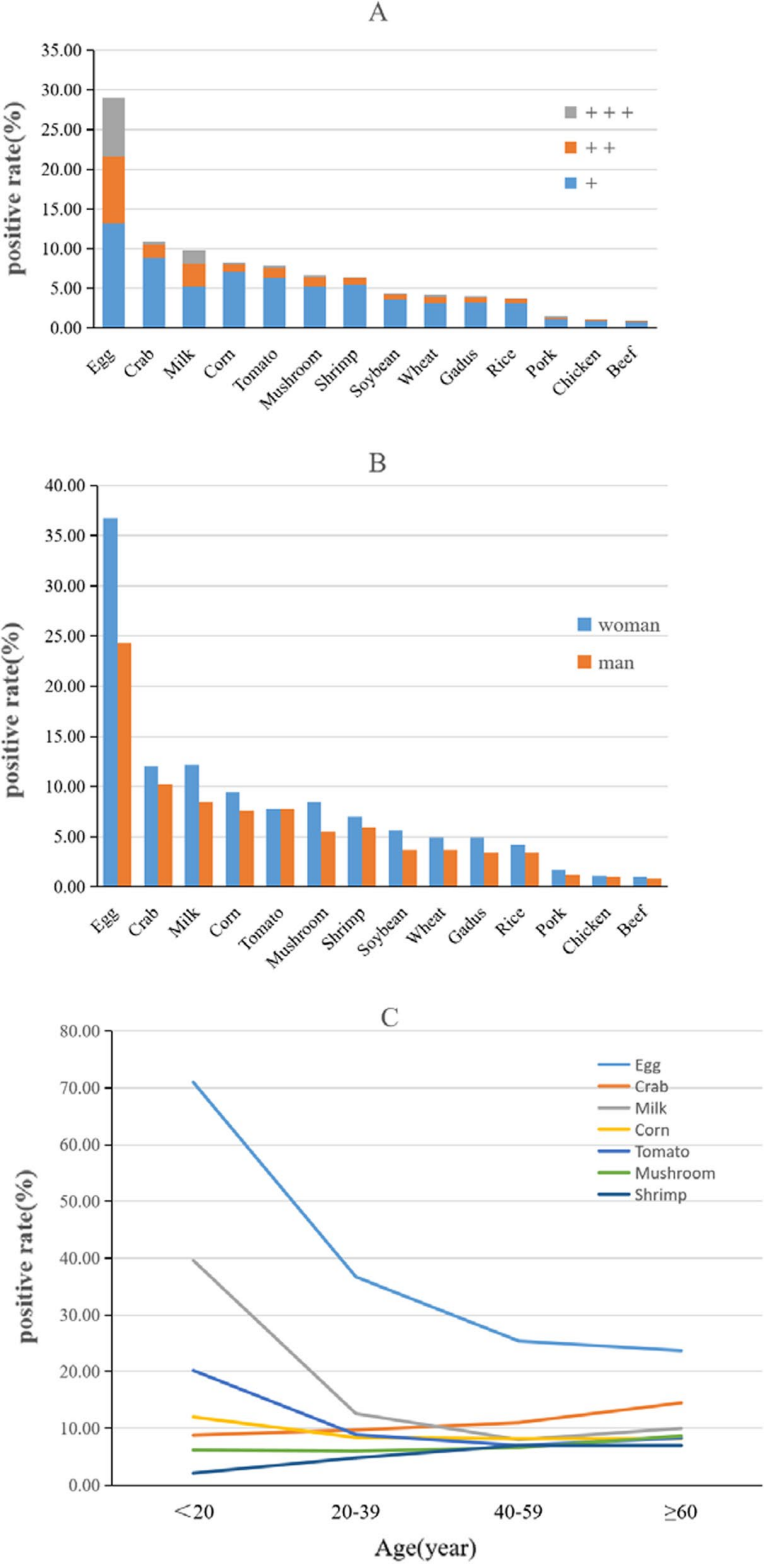
Distribution of food-specific IgG in the APEC. (**A** The positive rate of 14 kinds of common food. +: mildly positive, ++: moderately positive, + ++: severely positive; **B** The positive status of food-specific IgG in different sex; **C** The Distribution of food-specific IgG positive rate in different age groups (seven foods with higher positive rate)

The overall IgG positivity rate was up to 52.30%. The top seven IgG (+) foods were egg (29.01%), crab (10.89%), milk (9.84%), corn (8.28%), tomato (7.81%), mushrooms (6.62%) and shrimp (6.33%). The prevalence of severe and moderate positivity was higher in women than in men (12.92% vs. 7.40%, x^2^ = 518.61, *P* < 0.001; 29.66% vs. 20.90%, x^2^ = 464.06, *P* < 0.001). Younger probands were more likely to be IgG positive for egg, milk and tomato, but the opposite was true for crab, mushrooms and shrimp (Fig. 2).

### Analysis of physical examination data among different subgroups

In the ≥ 40 year-old group, IgG (+) group had a lower prevalence of overweight, central obesity, dyslipidemia, hypertriglyceridemia and hypercholesterolemia than the IgG ( −) group. There was no significant difference in the prevalence of high density lipoproteinemia between the two groups (24.83% vs. 24.78%, x^2^ = 0.01, *P* = 0.94). Furthermore, the IgG (+) group had a lower prevalence of abnormal blood glucose (16.23% vs. 18.67%, x^2^ = 29.34, *P* < 0.001), abnormal liver function (30.92% vs. 36.73%, x^2^ = 74.86, *P* < 0.001) and lower levels of UA and HB, while there was no difference in ALB levels between the two groups (45.47 ± 2.73 g/L vs. 45.52 ± 2.71 g/L, t = 1.34, *P* = 0.18).

Abdominal color Doppler ultrasonography showed that the prevalence of fatty liver was lower in the IgG (+) group (34.52% vs. 39.38%, x^2^ = 50.53, *P* < 0.001), but the prevalence of cholecystolithiasis, gallbladder polyps and renal calculi were not significantly different between the groups. Compared with the IgG (-) group, the IgG (+) group had a higher prevalence of thyroid disease (43.45 vs. 39.67%, x^2^ = 20.93, *P* < 0.001). The results of female breast color Doppler ultrasonography, gynecological ultrasonography and cervical cancer screening, as well as male prostatic nodule screening, showed that there was no significant difference between the two groups. The trend and distribution of the above indicators was similar in the < 40 year-old group (Table 1).

### Associations between food-specific IgG and health outcomes

The above analyses showed that the IgG (+) group had a lower prevalence of overweight, central obesity, abnormal blood lipids (including hypertriglyceridemia and hypercholesterolemia), abnormal FBG, abnormal liver function, and fatty liver than the IgG (-) group, as well as a higher prevalence of thyroid disease. Multivariate logistic regression analysis showed that after adjusting age, sex, body mass index, waistline, blood pressure, blood lipid and FBG, IgG positive state (vs. IgG negative) was associated with a lower prevalence of overweight (OR 0.92, 95% CI 0.87–0.96), FBG (OR 0.93, 95% CI 0.87–0.99) and TG (OR 0.87, 95%CI 0.83–0.92). In addition, IgG positive state was not significantly associated with abnormal SBP (OR 0.98, 95% CI 0.91–1.05), DPB (OR 0.93, 95% CI 0.87–1.01), or fatty liver (OR 0.98, 95% CI 0.92–1.04), but IgG positive subjects had a higher prevalence of thyroid disease (OR 1.09, 95% CI 1.04–1.15) (Table 2).

**Table 2:** Regression analysis of food-specific IgG and various healthy outcomes

Health outcomes	Odds ratio (95% CI))	*p* value	Adjusted odds ratio	*p* value
			(95% CI))	
Overweight	0.81 (0.78–0.85)	< 0.001	0.92 (0.87–0.96)	< 0.001
Central obesity	0.87 (0.83–0.91)	< 0.001	0.99 (0.94–1.04)	0.59
Dyslipidemia	0.86 (0.82–0.90)	< 0.001	0.96 (0.91–1.0)	0.07
Hypercholesterolemia	0.89 (0.85–0.94)	< 0.001	0.95 (0.90–1.0)	0.07
Hypertriglyceridemia	0.79 (0.75–0.83)	< 0.001	0.87 (0.83–0.92)	< 0.001
Low HDL-C	0.90 (0.81–1.0)	0.04	0.98 (0.88–1.09)	0.65
Pathoglycemia	0.83 (0.77–0.89)	< 0.001	0.93 (0.87–0.99)	0.03
Hyperuricemia	0.91 (0.86–0.96)	0.001	1.06 (0.99–1.13)	0.1
Dysarteriotony	0.86 (0.81–0.91)	< 0.001	0.98 (0.92–1.04)	0.47
Abnormal SBP	0.87 (0.81–0.93)	< 0.001	098 (0.91–1.05)	0.53
Abnormal DBP	0.83 (0.87–0.88)	< 0.001	0.93 (0.87–1.0)	0.05
Anemia	1.23 (1.11–1.45)	0.001	1.15 (1.01–1.33)	0.05
Abnormal liver function	0.85 (0.81–0.89)	< 0.001	0.98 (0.92–1.04)	0.47
Fatty liver disease	0.83 (0.79–0.81)	< 0.001	0.96 (0.90–1.03)	0.28
Thyroid disease	1.10 (1.05–1.16)	< 0.001	1.09 (1.04–1.15)	0.001

Adjusted for age, sex, BMI (except BMI-specific models), waistline (except waistline-specific models), blood pressure (except BP-specific models), blood lipid (except blood lipid specific models), FBG (except FBG-specific models). Fatty liver and uric acid were also introduced as covariates in the model with liver function as outcomes. Complete covariate data available for 10,637 person examinations

CI, confidence interval; BP, blood pressure; HDL-C, high density lipoprotein cholesterol; SBP, Systolic blood pressure; DBP, Diastolic blood pressure; FBG, fasting blood-glucose

### Further analysis of the relationships of food-specific IgG with TG, FBG and BMI

**Fig. 3:**
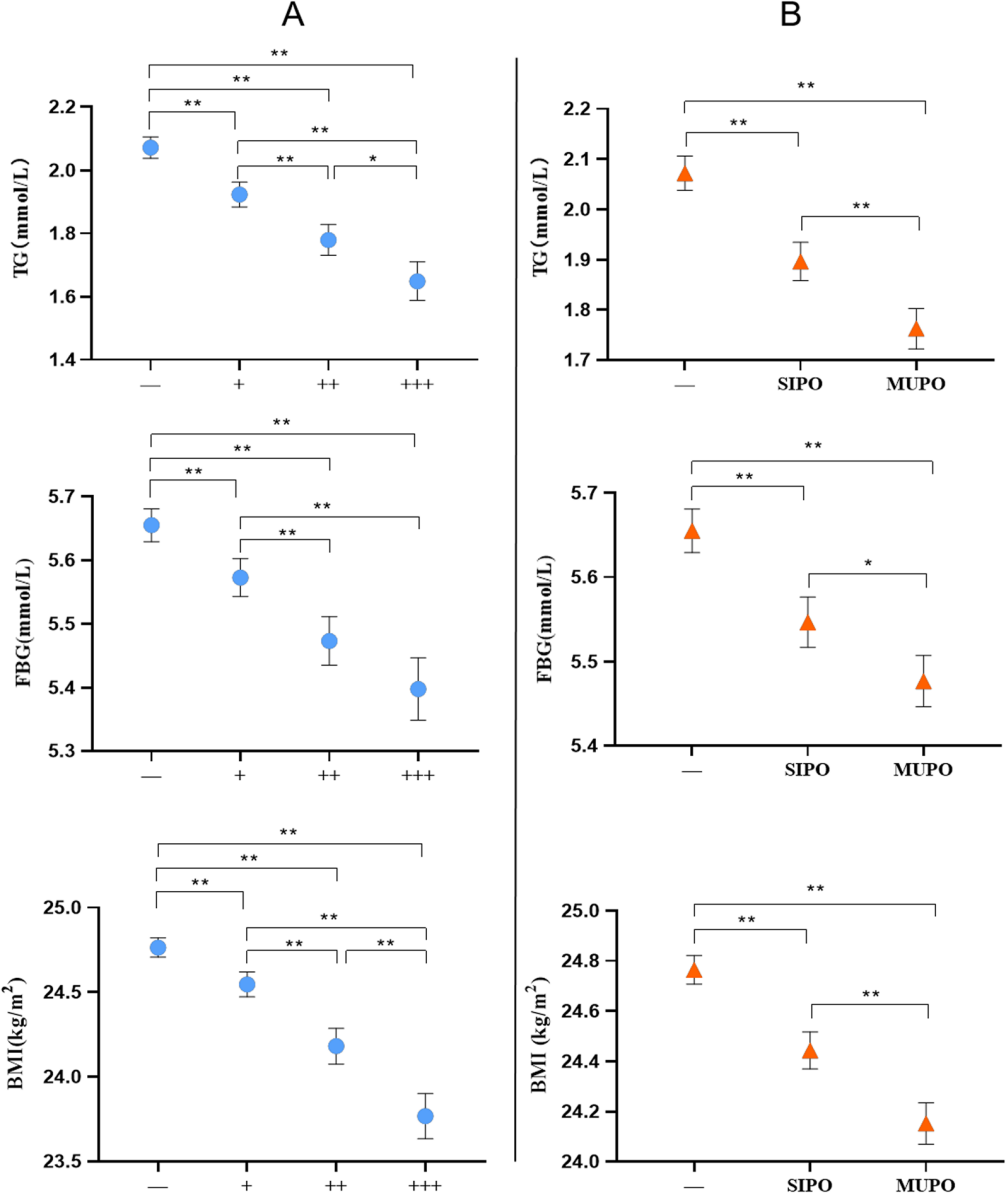
The distribution of TG, FBG and BMI in different groups. (Median and 95% CI. **A** The distribution of TG, FBG and BMI in different IgG titers. **B** The distribution of TG, FBG and BMI in different number of IgG positive foods. −: negative, +: mildly positive, ++: moderately positive, +++: severely positive. SIPO: single food specific IgG positive, MUPO: multiple foods specific IgG positive. BMI, body mass index; FBG, fasting blood-glucose; TG, triglycerides. * means *P* < 0.05 and ** means *P* < 0.001.)

The above analysis showed that food-specific IgG positive state was associated with a lower prevalence of abnormal FBG, hypertriglyceridemia and overweight. We further investigated the relationships of the IgG titers (−, +, ++, +++) and the number of IgG positive foods (−, SIPO and MUPO) with plasma FBG, TG and BMI. There were statistical differences in the distribution of TG, FBG and BMI (H = 372.30, *P* < 0.001; H = 216.67, *P* < 0.001; H = 243.33, *P* < 0.001) among the subgroups with different IgG titers, as well as in the SIPO and MUPO groups (H = 372.30, *P* < 0.001; H = 172.39, *P* < 0.001; H = 312.58, *P* < 0.001) (Fig. 3).

## Discussion

Different from previous studies focusing on patients with a specific disease, this study investigated an APEC with no apparent clinical symptoms and no special dietary guidelines. By analyzing food-specific IgG and physical examination results of the APEC, we deduced the distribution profile of food-specific IgG in the APEC and a possible correlation of food-specific IgG-mediated immune response with a number of health outcomes.

The positivity rate of food-specific IgG in the blood of the probands was up to 52.30%, generally with mild to moderate elevation of antibody titers, which was lower than that of ulcerative colitis patients (57.5–70.1%), Crohn disease patients (90.72%) [12, 21, 24], and that of food-specific IgGs associated with clinical allergic symptoms [25]. Such differences indicate that food-specific IgG with a higher positivity rate and a greater titer may be associated with specific pathological states, but it is not clear whether the disease results in an increase of the IgG positivity rate or the food-specific IgG causes the pathological state. In this study, we investigated 14 foods most frequently eaten by the Chinese population, and found a significantly higher IgG positivity rate in women than in men, which perhaps is somewhat associated with a greater prevalence of autoimmune disease (AI) in women than in men, as shown in previous studies [24, 26, 27].

The IgG positivity rate for some foods (e.g., crab, mushrooms and shrimp) was higher in older age, while a previous study found a negative correlation of IgG levels against all foods with age [28]. Another study showed that in Northeast China, a higher food-specific IgG positivity rate was observed for crabs, shrimp, eggs, milk and fish [29], which was greatly different from our study, which indicated that the food-specific IgG positivity rate was higher for eggs, crab, milk, corn and tomato. This suggests that the local dietary habits may influence the type of food-specific IgG prevalent in a specific area, which was avoided effectively because a large sample of the same area was used in this study.

It is believed that food-specific IgG is involved in the body’s complex immune response [29]. Some foods cannot be completely digested by the human body due to a lack of corresponding enzymes, and they are recognized as foreign substances in the gastrointestinal mucosal lymphatic tissues, leading to the formation of immune complexes with specific IgG antibodies in the body. Subsequently, macromolecular complexes are phagocytized by monocytes, while micromolecular complexes are excreted by the kidneys. It remains unknown whether such an IgG-mediated immune response has broad effects on the physical examination results of healthy probands. This study showed no difference in the prevalence of abnormal renal function between the two groups. In the IgG (-) group, renal disfunction seemed to have a lower trend and the prevalence of fatty liver was much lower, but there was no association between these characteristics with the IgG positive state after adjusting for confounding factors reported in the literature. Furthermore, there were significant differences in the prevalence of health outcomes (including abnormal blood pressure, hyperuricemia) between the two groups (Table 1), but the IgG positive state had no statistically significant influence on these health outcomes after adjusting for age, sex and other reported confounding factors.

Previous studies revealed that food-specific IgG may affect the body’s nutritional status [12], but no difference in ALB was found between the two groups in this study. Although the prevalence of anemia was higher in the IgG (+) group, there was no association between food-specific IgG and anemia after excluding factors such as age and sex. Food-specific IgG positive state was related with a lower risk of hypertriglyceridemia, abnormal FBG and overweight, suggesting that it may be an independent protecting factor for these indicators and it was more closely associated at a higher titer and in MUPO probands (Fig. 3). To our best knowledge, this phenomenon was firstly observed in this APEC in humans, but it is similar to the results of an in vivo animal experiment by Batista et al. [30], who found a decrease in body weight, blood glucose and blood lipids, which was followed by a decline of plasma IgE levels, a gradual elevation of IgG levels, and a slight decrease of metabolic indicators (as compared with at the allergic state) in mice treated with sensibiligens after continuing the food antigen stimulation. These similar metabolic characteristics suggest that food-specific IgG is somewhat associated with food allergy.

Acute food allergy and chronic food allergy are correlated with a set of co-factors [31]. However, the diagnosis of food allergy is a clinical challenge and requires history inquiry and food exclusion in most cases [32, 33]. Moreover, the diagnosis is very difficult to make in patients without apparent symptoms. In our study, IgG positive subjects might initially be in a non-obvious food allergy state after contacting some foods, and no restrictions are imposed on food intake in daily life due to an absence of significant symptoms. Similarly, there was long-time repeated food antigen stimulation, which induced immunotolerance. Thus, food-specific IgG with corresponding metabolic changes was detected in the body of these subjects. This is in agreement with the view that food-specific IgG confers natural protection against food allergy [34]. IgG can send negative signals after binding to the FcgRIIb receptor of mast cells, and can also sterically block the binding of IgE to mast cells, thereby suppressing the allergic reaction [15]. These effects of IgG have been confirmed in clinical studies. For example, Savilahti et al. found that milk tolerance in allergic children was associated with blood IgG levels [35]. In addition, maternal food-specific IgG may be correlated with the food tolerance of offspring [36].

Finally, our study also has some potential limitations. First, we did not have specific information about the diet of the subjects, which was associated with overweight [37]. Second, we lacked data related to family history of allergy, so these variables could not be included in multivariate logistic regression. Finally, this study was observational and retrospective in design, which prevented us from being able to draw conclusions about causal relationships between food-specific IgG and health outcomes. Therefore, larger prospective studies are required to establish these relationships.

## Conclusions

Food-specific IgG was widely present in APEC, predominantly with mild to moderate elevation. We showed that food-specific IgG is associated with a normal body weight and metabolic indicators (mainly TG and FBG). Therefore, dietary guidelines based on food-specific IgG may be helpful for people with overweight or metabolic disorders. The underlying physiological mechanism merits further study.

## Data Availability

The datasets used and/or analysed during the current study are available from the corresponding author on reasonable request.

## Abbreviations

APEC: Asymptomatic physical examination cohort
FBG: Fasting blood glucose
BMI: Body mass index
BP: Blood pressure
SBP: Systolic blood pressure
DBP: Diastolic blood pressure
LDL-C: Low density lipoprotein cholesterol
HDL-C: High density lipoprotein cholesterol
TC: Total cholesterol
TG: Triglycerides
UA: Uric acid
ALB: Plasma albumin
HB: Hemoglobin
Cr: Creatinine
CI: Confidence interval
OR: Odds ratio
SIPO: Single food-specific positive
MUPO: Multiple food-specific IgG positive
